# Development of an in vitro model to measure bioactivity of botulinum neurotoxin A in rat bladder muscle strips

**DOI:** 10.1186/1471-2490-14-37

**Published:** 2014-05-15

**Authors:** Janneke IM van Uhm, Goedele MA Beckers, Willem J van der Laarse, Eric JH Meuleman, Albert A Geldof, Jakko A Nieuwenhuijzen

**Affiliations:** 1 Department of Urology, VU University Medical Center, PO Box 7057, Amsterdam 1007 MB, The Netherlands; 2 Department of Physiology, VU University Medical Center, Amsterdam, The Netherlands; 3 Department of Radiology and Nuclear Medicine, VU University Medical Center, Amsterdam, The Netherlands

**Keywords:** Bladder, Botulinum toxin, Type A, In vitro

## Abstract

**Background:**

Botulinum toxin A (BoNT-A) is a new treatment modality in various causes of bladder dysfunction; like neurogenic detrusor overactivity and overactive bladder. The best technique of administrating BoNT-A in patients is unknown. A validated in vitro model could be used to investigate newer intravesical administration techniques of BoNT-A. In this study, we describe the development and validation of in vitro model to measure inhibitory effects of BoNT-A on bladder strip contractions.

**Methods:**

Rat bladder strips were mounted in organ baths filled with Krebs’ solution. The strips were stimulated chemically (80 mM potassium chloride, 1 μM carbachol) and electrically (Electrical Field Stimulation (EFS) 100 shocks, 50 V, 20 Hz, every 3 minutes). The viability of the strips was measured by carbachol stimulation at the beginning and at the end of the experiments. The strips were incubated in various concentrations of BoNT-A (0.03, 0.2, 0.3 nM). Controls were incubated in Krebs’ solution only. The inhibition of strip contraction induced by EFS was measured. These measurements were statistically analyzed with a log-logistic model representing diffusion.

**Results:**

All strips remained viable during the experiments. Inhibition of strip contraction was observed after incubation with 0.3 nM BoNT-A. The measurements fitted to a log-logistic model describing diffusion of BoNT-A in the bladder strip. The parameters of the log-logistic model representing diffusion were significant for 0.3 nM BoNT-A. Incubation with 0.2 nM BoNT-A showed insignificant results for 2 out of 3 runs. Incubation with 0.03 nM BoNT-A did not result in significant inhibition of strip contractions.

**Conclusions:**

An in vitro model was developed and validated in which the inhibitory effect of low concentrations of BoNT-A on bladder strip contractions can be measured.

## Background

Botulinum neurotoxin A (BoNT-A) was introduced for the treatment of overactive bladder (OAB) by Schurch et al. at the beginning of this century [1]. Since then, multiple clinical trials proved that BoNT-A is an effective therapy for the treatment of refractory OAB [2]. However, these clinical trials demonstrated differences in efficacy and duration of the response. The most effective doses, volumes of dilution, numbers of injection and the best sites of injections needed for effective treatment are not well known [3]. Despite evolving knowledge about the mechanism of action of BoNT-A on neurotransmitter release and receptor activation, the spread of BoNT-A after intravesical injections remains unknown [4,5]. If we would be able to monitor the spread of BoNT-A in the bladder wall after administration, we might understand the differences in clinical effect in patients. Furthermore, we could develop the most favourable method of administrating BoNT-A in the bladder.

To gain insight into the mechanism of spread of BoNT-A after injection in the bladder wall, we would need a reliable in vitro model with reproducible results. Smith et al. were the first to measure bioactivity of BoNT-A in an in vitro model with rat bladder strips [6]. Howles et al. on the other hand could not reproduce these measurements in another in vitro model [7]. Takahashi et al. demonstrated inhibitory effect of BoNT-A in vitro however it was at a much higher concentration (>10 nM) than is used in clinical practice (10 U/1 mL onabotulinumtoxinA = 0.001 nM) [8]. In this study, we further developed and validated an in vitro model to measure the bioactivity of BoNT-A in lower concentrations. To reach this goal, we sought for the best mathematical model to better understand the spread of BoNT-A in a bladder strip.

## Methods

### Tissue preparation and strip tension measurement

Eight adult male Wistar rats (250–300 g, Harlan, The Netherlands) were used. All experiments were performed in accordance with institutional guidelines and were approved by the ethics committee for animal experiments of the VU University Amsterdam. The urinary bladder was removed following isoflurane anaesthesia and heart dissection of the rat. The bladder was immediately placed in Krebs’ solution (mmol/L; NaCl 113, KCl 4.8, MgSO_4_ 1.2, KH_2_PO_4_ 1.2, CaCl_2_*2H_2_O 2.5, NaHCO_3_ 25, glucose 11.5, bubbled with 95% O_2_ and 5% CO_2_ to attain a pH of 7.4) at 35 degrees Celsius.

**Figure 1:**
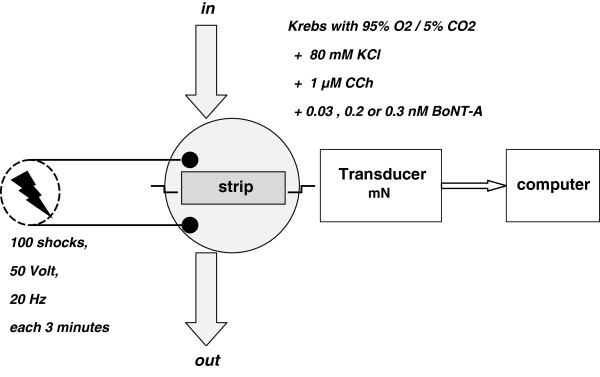
**Schematic representation of the in vitro model.** The organ bath was continuously perfused with Krebs’ solution. Chemical stimulation was performed by perfusion the bath with 80 mM KCl or 1 μM CCh and flushing the bath with Krebs’ solution in between. During BoNT-A incubation, the bath was continuously perfused by the same concentration of BoNT-A (0.03, 0.2, 0.3 nM).

Two longitudinal strips of maximally 0.7 by 7 mm were cut from the dorsal side of the bladder body under a microscope. The urothelial layer of the strips was meticulously removed with micro scissors under dark field illumination. The serosal layer of the bladder was not removed.The two rat bladder strips were mounted between 2 platinum hooks in two parallel organ baths. One of the platinum hooks was connected directly to a force transducer (AE801, SensoNor, Norway) (Figure 1). The baths were perfused with 35°C Krebs’ solution for 60 minutes to stabilize the strips at a passive tension of 10 millinewton. Hereafter the bladder strips were stimulated. Chemical stimulation was performed with 80 mM potassium chloride (KCl) or 1 μM carbachol (CCh). Between the chemical stimulations and during the electrical field stimulation, the organ baths were perfused by Krebs’ solution. Electrical field stimulation (EFS) between two platinum plate electrodes was delivered by a Grass SD9 stimulator. The intrinsic nerves were stimulated with 50 V pulses of 100 shocks at a frequency of 20 Hz. The stimulation interval was 3 minutes.

Before we started the BoNT-A experiments, we incubated two strips in 1 μM tetrodotoxin (TTX) during continuous EFS to verify that the EFS protocol induced primarily nerve cell depolarization [9]. Subsequently, we incubated two strips in Krebs’ solution and gradually lowered the oxygen tension in the organ bath from 95% to 20%. We calculated the critical oxygen tension in the organ bath by a Hill diffusion model previously described by Van der Laarse et al. [10]. Contractions were recorded by a data acquisition program (NEWXLC® software developed by the Department of Physiology, VUmc, Amsterdam).

### Drugs and solutions

KCl, CCh, TTX and BoNT-A were obtained from Sigma-Aldrich, Zwijndrecht, The Netherlands. The BoNT-A used was pure BoNT-A (molecular weight of 150 kDa), i.e. toxin without any enveloping proteins. KCL, CCh, TTX and BoNT-A were all dissolved in Krebs’ solution. All solutions were gassed with a mixture of 95% O_2_ and 5% CO_2_ (pH 7.4 at 35°C).

### Viability of bladder strips

To establish the viability of the bladder strips after preparation and during the stimulation protocol, the strips were excited chemically by adding 80 mM KCl and 1 μM CCh to the organ baths. The organ baths were flushed with Krebs’ solution after chemical stimulation. KCl induces smooth muscle membrane depolarization without the need of activation of any receptor or second messenger system [9]. CCh induces contraction of the bladder strip by direct activation of the muscarinic receptor at the smooth muscle cell membrane. CCh interacts with the same muscarinic receptor as acetylcholine [9]. BoNT-A inhibits the release of acetylcholine and it does not interact with the muscarinic receptor at the muscle cell membrane. CCh still induces strip contraction after BoNT-A incubation. The comparison of CCh-induced strip contraction before and after BoNT-A incubation acts as a viability test of this strip during the experiment.

### Stimulation protocol

KCl and CCh were used to verify viability, while EFS provided baseline contractions during phase 1. Organ baths were perfused with 80 mM KCl for 10 seconds, followed by flushing the organ baths with Krebs’ solution for 3 minutes. This cycle was performed three times. Subsequently, organ baths were perfused 3 times with 1 μM CCh solution followed by flushing the organ baths with Krebs’ solution for 3 minutes. Finally, contractions were induced by EFS (50 V, 20 Hz, 100 shocks) for five times, each with an interval of 3 minutes.

In phase 2, strips were incubated in various concentrations BoNT-A (0.03, 0.2, 0.3 nM). Control strips were perfused by Krebs’ solution only. Repetitive EFS was performed.

Phase 3 consisted of perfusion of Krebs’ solution in the organ baths for at least 5 minutes. Subsequently, repetitive EFS and CCh stimulations with the same settings and the same concentrations as used in phase 1 were performed. We refer further to a completed protocol of stimulations as a ‘run’.

### BoNT-A incubation

Each BoNT-A concentration was tested 3 times. Two strips of the same bladder were incubated in 2 parallel organ baths. One strip was incubated in 0.3 nM BoNT-A (run 1a, 2a, 4a) whereas the other strip was perfused with Krebs’ solution (=control: run 1b, 2b, 3b). To establish whether there was a minimum concentration necessary to measure an inhibitory effect of BoNT-A in bladder strip contractions, run 3a, 4b and 5a contained strips incubated in 0.2 nM BoNT-A. Run 5b, 6a, 6b contained strips incubated in BoNT-A 0.03 nM.

### Data analysis

Three peak forces of strip contraction induced by CCh stimulation were measured and averaged (*mPF CCh*). The viability of the strip was established by the equation: *Contractility = mPF CCh phase III/mPF CCh phase I*. A strip is viable if contractility equals 1.

Inhibition of bladder strip contraction by BoNT-A was measured by comparing the mean peak force induced by EFS before and during BoNT-A incubation. Inhibition is defined by: *Inhibition = 1 – (mPF incub/mPF max),* in which *mPF incub* is the mean of five repetitive peak forces by EFS measured every 30 minutes during incubation, and *mPF max* is the mean of five highest repetitive peak forces of strip contractions induced by EFS at the start of phase II.

We analyzed the data by a mathematical model representing diffusion, as we hypothesized that BoNT-A spreads in the bladder strip by diffusion. The model is a log-logistic model [11]: *I = I*_*max*_/(1 + exp (- b_*_ln (t/m))), where I = measured inhibition of contraction of bladder strip, I_max_ = calculated maximum of inhibition, b = shape parameter of the curve, t = time after highest peak forces of EFS at start of incubation (hours), and m = mid inhibition time, at which I = 0.5 I_max_ (hours). R^2^ is predictive accuracy of the model, so the closer R^2^ is to 1.0, the better the data fit to the curve. The curve fits if R^2^ is more than 0.95, and if the 95% confidence intervals around b and m are significant. Data analysis and curve fitting by non-linear regression was done with SPSS 20®.

## Results

### Viability of bladder strips

Strip contraction induced by EFS decreased by more than 90% after 1 μM TTX incubation. The oxygen tension in the organ bath was about 700 mmHg (pO2 95%) and the critical oxygen tension during EFS was about 600 mmHg (pO2 80%) with a diameter of the strip of maximally 0.7 mm.

All bladder strips contracted during perfusion of 80 mM KCl at the start of the runs. Median contractility *(mPF CCh phase III/mPF CCh phase I)* of all strips was 1.1 (range 0.9 – 1.4). One strip (run 6b) was lost due to technical problems.

### BoNT-A effect on strip contractions

**Figure 2:**
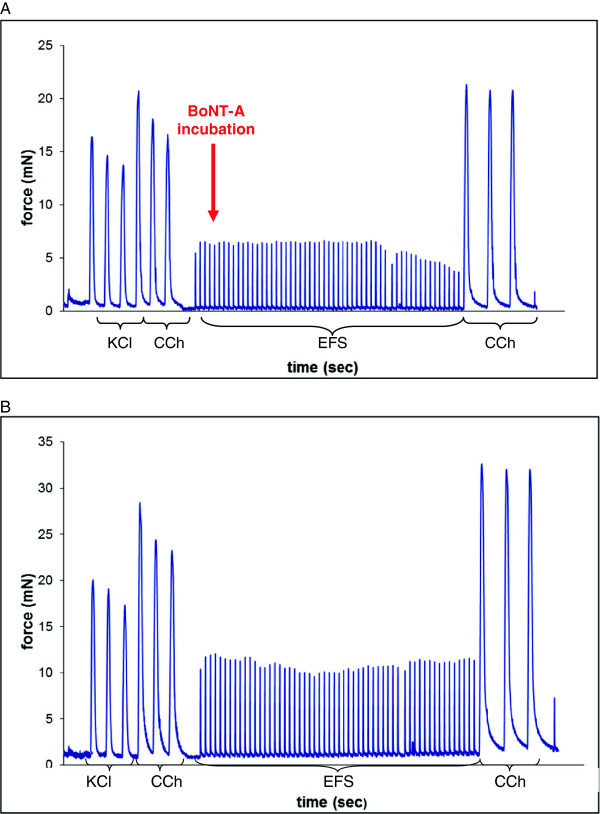
**A-B: Bladder strip contractions after BoNT-A incubation. A**: Response of bladder strip contraction force (mN) to KCl, CCh, EFS before and after 0.3 nM BoNT-A incubation (run 1a). **B**: Response of bladder strip contraction force (mN) to KCl, CCh and EFS during Krebs perfusion (run 1b = control).

**Figure 3:**
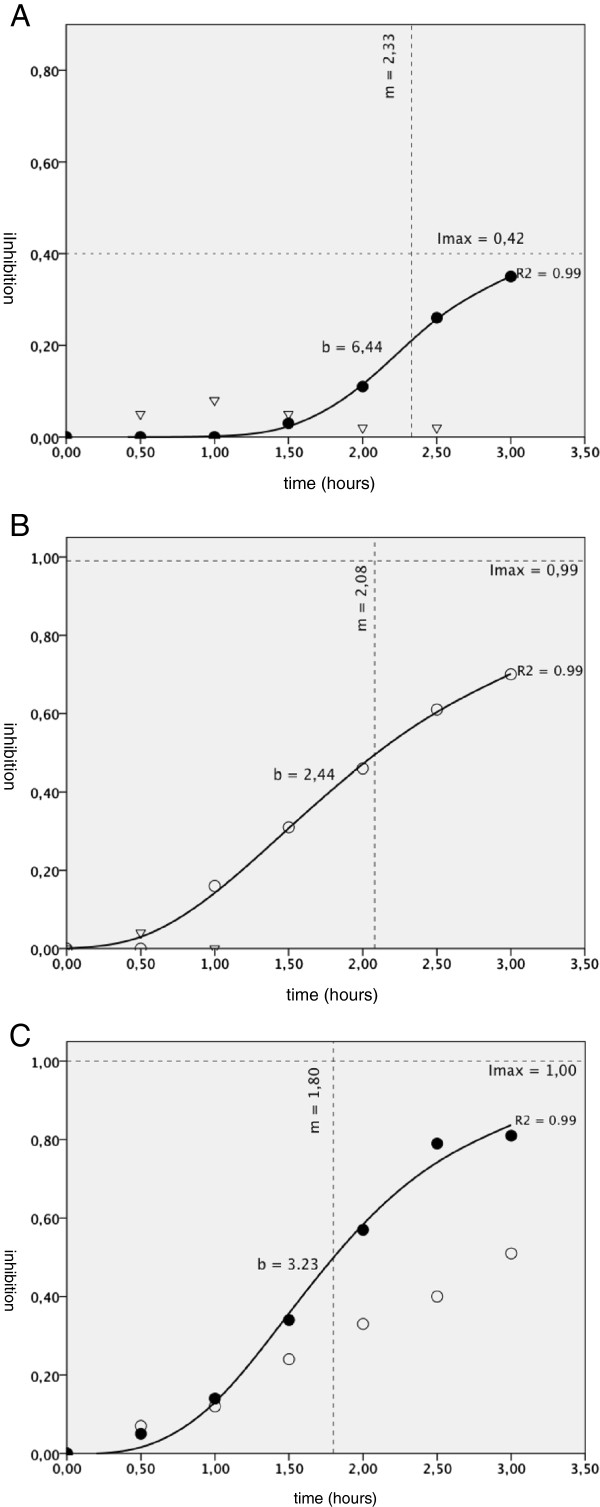
**A-C: Measured inhibition of bladder strip contraction and fitted curves.** Measured inhibition of bladder strip contraction after incubation in 0 nM (control), 0.2 nM and 0.3 nM BoNT-A in time (hours) with the fitted curves. I_max_ = calculated maximum of inhibition, b = shape parameter of the curve, t = time after highest peak forces of EFS at start of incubation (hours), m = mid inhibition time (hours), R^2^ = predictive accuracy of the fitted curve. **A** = run 1 ∇ = 0 nM (control), ● = 0.3 nM BoNT-A. **B** = run 3 ∇ = 0 nM (control), O = 0.2 nM BoNT-A. **C** = run 4 O = 0.2 nM BoNT-A, ● = 0.3 nM BoNT-A.

After incubation of strips in 0.3 nM BoNT-A, the Imax was 0.42, 0.62 and 1.00 for run 1a, 2a and 4a respectively (Table 1, Figures 2 and 3). R^2^ was 0.99. Respective mid inhibition times (m) were 2.33 (95% C.I. = 2.22-2.44), 1.49 (95% C.I. = 1.18-1.80) and 1.80 (95% C.I. = 1.31-2.28) hours. Shape parameters of the curve (b) were 6.44 (95% C.I. = 5.33-7.54), 3.35 (95% C.I. = 1.90-4.79) and 3.23 (95% C.I. = 1.55-4.90).

**Table 1:** The results of maximum inhibition of strip contraction calculated by a log-logistic model which represents diffusion

**BoNT-A (nM)**	**run**	**I**_**max**_	**CI**	**m**	**CI**	**b**	**CI**	**R**^**2**^
0.3	1a	0.42	0.37-0.47	2.33	2.22-2.44	6.44	5.33-7.54	0.99
0.3	2a	0.62	0.46-0.77	1.49	1.18-1.80	3.35	1.90-4.79	0.99
0.3	4a	1.00	0.66-1.33	1.80	1.31-2.28	3.23	1.55-4.90	0.99
0.2	3a	0.99	0.61-1.37	2.08	1.33-2.82	2.44	1.54-3.33	0.99
0.2	4b	0.82	min 0.02- 1.65	2.43	min 0.36-5.22	1.83	0.59-3.08	0.99
0.2	5a	1.00	min 0.25-2.25	3.26	0.51-6.01	2.96	0.89-5.03	0.98
0.03	5b	0.06	0.05-0.08	1.19	0.87-1.50	4.45	min 0.42-9.31	0.96
0.03	6a	0.83	min 8.04-9.71	7.51	min 49.76-64.79	2.02	min 1.25-5.30	0.89
0	1b	0.02	min 17.7-17.7	0.52	min 3304-3305	0.32	min 420-420	•
0	2b	0.16	0.03-0.29	0.24	min 1.38-1.87	1.83	min 18.0-21.7	0.35
0	3b	0.01	min 19.4-19.4	2.70	min 2067-2067	3.01	min 978-985	•

The data with 95% confidence interval (CI) of calculated maximum of inhibition (I_max_), mid inhibition time in hours (m), shape parameter of the curve (b), predictive accuracy of the model (R^2^) of various BoNT-A concentrations during run 1 to 6. R^2^ could not be calculated for 0 nM during run 1b and run 3b.

Incubation of strips in 0.2 nM BoNT-A solution during run 3a, 4b and 5a showed incompatible results. Run 3a demonstrated a good fit of the curve with R^2^ = 0.99, m = 2.08 (95% C.I. = 1.33-2.82) and b = 2.44 (95% C.I. = 1.54-3.33). However, run 4b and 5a had an insignificant 95% C.I. of m and b although R^2^ was good. BoNT-A concentration of 0.03 nM did not induce inhibition of strip contraction during run 5b and 6a, I_max_ was respectively 0.06 (95% C.I. = 0.05-0.08) and 0.83 (95% C.I. = min 8.04 – 9.71). No inhibition of strip contractions was observed in the control runs (1b, 2b, 3b) using Krebs’ solution.

## Discussion

In this study, an in vitro bladder strip model was validated to measure the spread and the inhibitory effects of low concentrations of BoNT-A on bladder muscle contractions. The spread of BoNT-A in bladder strips could be reproduced and confirmed using a statistical model representing diffusion at a concentration of 0.3 nM. A lower concentration of 0.2 nM showed inhibition, however insignificant results for 2 out of 3 runs. The inhibitory effects of BoNT-A on bladder strip contractions could be demonstrated at lower concentrations than previously described.

Smith et al. were the first to demonstrate inhibition of bladder strips by BoNT-A in an in vitro model [6]. The model was based on various electrical field stimulation protocols during incubation of the strips with BoNT-A. Contractions of the strips did indeed decrease during incubation, but viability of the strips was not measured during the experiment. Howles et al. were unable to reproduce in vitro muscle strip inhibition by BoNT-A [6] In these experiments, the commercial products Botox® (onabotulinumtoxinA = 900 kD) and Dysport® (abobotulinumtoxinA = 500 kD) were used, in which BoNT-A is enveloped in large proteins [7]. These proteins might be too large to diffuse through the luminal or serosal surface of the bladder strips. Therefore we used pure BoNT-A and also for this reason we removed the bladder mucosa from the strip before mounting the strip in the organ bath, as did the group of Smith et al. [7].

To be sure that the EFS protocol primarily induces nerve cell depolarization, strips were incubated in TTX before applying EFS [9]. TTX selectively blocks the sodium-channels in nerve cell membranes which effectively inhibits nerve cell depolarization. Stimulation by EFS after TTX incubation resulted in an almost complete inhibition of strip contractions. In addition, we verified that the oxygen tension was not limiting strip contraction by keeping the oxygen tension in the organ bath higher than the critical oxygen tension [10].

We demonstrated that the inhibitory effect of BoNT-A at a concentration of 0.3 nM is the result of diffusion. Incubation of strips with 0.2 nM BoNT-A demonstrated equivocal diffusion results. Runs with lower concentrations (0.03 nM) demonstrated no inhibition. This suggested that there is a minimum BoNT-A concentration needed for bladder muscle strip inhibition in vitro. We demonstrated that the lowest effective concentration in this setting lies between 0.2 and 0.3 nM.

It is self-evident that continuous perfusion of BoNT-A in a bladder strip model is not directly comparable to intravesical injections in patients. The much higher concentration needed in vitro (0.3 nM) compared to the concentration needed in patients (onabotulinumtoxinA 10 U/mL = 0.001 nM) might be explained by the technique of administration. Moreover, the effect in vitro is measured within a few hours after administration due to susceptibility of the strip, whereas the effect of intravesical injections in patients becomes apparent after days or weeks [3].

Our study completed the model of Smith et al. and Takahashi et al. [6,8] by controlling the viability of the bladder strips during the runs. We proved that the inhibitory effect of BoNT-A can be measured in vitro at low concentrations. Furthermore, we postulated how BoNT-A reaches the nerve endings in the musculus detrusor after injection. The statistical analysis suggests that BoNT-A spreads through the muscle by diffusion. Our results were significant for the highest concentration used. For the lower concentration of 0.2 nM, curve fitting was good, but parameters were insignificant. The spread by diffusion can explain why BoNT-A acts best when injected at multiple sides into the bladder wall. This model can be used to study effectiveness of BoNT-A using other techniques of administration such as electromotive administration [12,13] or intravesical delivery using liposomes [14].

## Conclusions

In this study we describe an in vitro model in which the inhibitory effect of low concentration of BoNT-A on bladder strip contractions can be measured. This model can be used in the future to investigate newer intravesical administration techniques of BoNT-A.

## Abbreviations

OAB: Overactive bladder; BoNT-A: Botulinum neurotoxin A; KCl: Potassium chloride; CCh: Carbachol; EFS: Electrical field stimulation; TTX: Tetrodotoxin.

## Competing interests

JIMU, EJHM, AAG, GMAB and WJL have no competing interests. JAN is a consultant for Astellas and performed teaching courses for Coloplast, Allergan and AstraTech.

## Authors’ contributions

JIMU, EJHM and AAG participated in the conception of the study. The study is designed by JIMU, WJL and AAG. The data are collected by JIMU and WJL. The data are analyzed by JIMU, GMAB and JAN. The manuscript writing is done by JIMU, GMAB and JAN. The manuscript is critical revised by WJL, EJHM and AAG. All authors read and approved the final manuscript.

## Pre-publication history

The pre-publication history for this paper can be accessed here:

http://www.biomedcentral.com/1471-2490/14/37/prepub
